# Supplementary material for: Definition of germ layer cell lineage alternative splicing programs reveals a critical role for Quaking in specifying cardiac cell fate
Source: Nucleic Acids Res. 2022 May 11;50(9):5313–34. doi: 10.1093/nar/gkac327 (PMC9122611; doi:10.1093/nar/gkac327)
Supplement: gkac327_Supplemental_Files [file gkac327_supplemental_files.zip › LinSpecific-AS-QKI-Meso_SUPPL-Figs-Legends_REV.pdf]

SUPPLEMENTAL FIGURE 1: A. Fluorescence-activated cell sorting (FACS) strategy showing density plots for day 3 differentiated DE cells' antibody isotype control (right) and a single representative of three independent replicates for CXCR4:APC stained cells (right) that were selected by FACS based on GFP (y-axis) and CXCR4 (x-axis) dual positivity and from which RNA was extracted for RNA-seq (the cells constitutively express a nuclear localized humanized renilla GFP (hrGFP-NLS)). **B.** Indirect immunofluorescence of H9 hESCs with hrGFP-NLS, showing hrGFP (green), SOX2 (magenta), SSEA3 (yellow), and DAPI (blue; SOX2, SSEA3, and DAPI in overlay) for UD hESCs; hrGFP (green), SOX17 (magenta), and DAPI (blue; SOX17 and DAPI overlay) for DE cells, hrGFP (green); NKX2.5 (magenta), and DAPI (blue; NKX2.5 and DAPI overlay) for CM cells; and hrGFP (green), SOX2 (magenta), and DAPI (blue; SOX2 and DAPI overlay) for ECT cells (scale bar = 100 μm). **C.** Principal component analysis plot of the top 500 genes with the most variance between replicates of UD, DE, CM, and ECT cells, clustered by similarity (n = 3 each). **D.** Overlap of protein-coding transcript abundances that significantly changed in CM, DE, and ECT cells relative to UD hESCs from DESEQ2 analysis of RNA-seq data. **E.** Table showing total number of alternatively spliced events significantly changing in CM cells relative to UD (CM/UD), DE cells relative to UD (DE/UD), ECT cells relative to UD (ECT/UD), or CM cells relative to DE cells (CM/DE) by VAST-tools analysis of RNA-seq data. **F.** Change in percent retained intron (dPRI) for intron retention events (positive values indicate an increase in intron retention, lower values indicate a decrease) in DE, CM, and ECT cells, significantly changing relative to UD hESCs in violin plot, and overlap of these shown in Venn diagram inset (ns = not significant, and ****$P \leq 0.001$ by Mann-Whitney test). **G.** Heatmap showing hierarchical clustering of significantly changing retained introns from DE, CM, and ECT cells, calculated by dPRI relative to UD hESCs. **H.** Comparison of dPSI values calculated by RT-PCR and BioAnalyzer quantitation (y-axis) versus VAST-tools analysis of RNA-seq (x-axis) data for each alternatively spliced exon shown in Fig 1E; R^2 values are shown for DE cells compared to UD hESCs (DErelUD), CM cells compared to UD hESCs (CMrelUD), ECT cells compared to UD hESCs (ECTrelUD), and CM cells compared to DE cells (CMrelDE) in the inset. **I.** dPRI of significantly changing retained introns for CM cells relative to DE cells shown in violin plot, and the overlap of these compared to CM cells relative to UD hESCs and DE cells relative to UD hESCs shown in the Venn diagram inset, calculated from VAST-tools analysis of RNA-seq data. **J.** Dot plot comparing dPSI values obtained by VAST-tools (y-axis) to dPSI values obtained by rMATS for CE/MIC events indicating those

significant in rMATS (red), VAST-tools (blue), both (orange), or significant in both but in opposite directions (Opposing sign; green), or neither (open circle) for CM/UD, DE/UD, ECT/UD, or CM/DE.

SUPPLEMENTAL FIGURE 2: A. Summary of QKI binding frequency to lineage-specific alternatively spliced exons identified by both VAST-tools and rMATS analysis. “Cell” denotes cell type in which CLIP experiment was performed (@1 refers to experimental replicate number one; @2 refers to experimental replicate number two), “RBP” is the RBP of interest (QKI), “Overlap” is the number of occurrences in which merged coordinates of interest (upstream intron, alternatively spliced exon, and downstream intron) intersect with at least one QKI CLIP peak, “Total” is the total number of cassette exons that passed significance cutoff in both VAST-tools and rMATS, and “Ratio” is the ratio of overlap to total (values in bold are the highest observed for all “Cell” conditions), which measures the ratio of QKI binding to cassette exons and flanking intron sequence. CEs that are more included in CM cells relative to DE cells are shown in blue, more skipped CEs are shown in yellow, with the sum of events below. **B.** UCSC Genome Browser screen shots showing QKI eCLIP peaks, Gencode Gene annotation for *CLSTN1, PICALM, SLK,* and *MARK3*, with RNA-seq coverage tracks for UD hESCs (black), DE cells (magenta), CM cells (cyan), and ECT cells (yellow), and conservation of 100 vertebrates track. **C.** Heatmap showing hierarchical clustering of annotated RBPs that change significantly in at least one lineage by DESEQ2 analysis of RNA-seq data; $\log_2$ fold change values from CM cells relative to DE cells, DE cells relative to UD hESCs, CM cells relative to UD hESCs, and ECT cells relative to UD hESCs were used to make the heatmap and are displayed as row Z-score values. **D.** Heatmap showing hierarchical clustering of annotated RBPs that change significantly in at least one lineage by LC-MS/MS analysis; $\log_2$ fold change values from CM cells relative to DE cells, CM cells relative to UD hESCs, and DE cells relative to UD hESCs were used to make the heatmap and are displayed as row Z-score values. **E.** Western blot of protein extracted from UD hESCs, DE cells, and CM cells probed with antibodies for QKI5 (magenta, top panel) and panQKI (green), QKI6 (magenta, middle panel) and panQKI (green), or QKI7 (magenta, bottom panel; ND denotes not detectable) and panQKI (green); quantitation of each isoform relative to total panQKI is shown below by bar graph. **F.** Indirect immunofluorescence of H9 hrGFP-NLS showing panQKI (magenta), SSEA3 (yellow), hrGFP (green), and each overlaid for UD hESCs; panQKI (magenta), SOX17 (yellow), and hrGFP (green), with each overlaid for DE cells; panQKI (magenta), NKX2.5 (yellow), and hrGFP (green), with each overlaid for CM cells (scalebar denotes 100µm).

SUPPLEMENTAL FIGURE 3: A. Chromatograms showing reads from genomic DNA amplified by PCR then cloned and sequenced from NKX2.5→GFP WT and QKI KO hESCs: the top panel shows WT sequence with guide RNA target noted in box. The next two panels show two independent sequences derived from polyclonal QKI KO cells (vertical box indicates nucleotide insertion); the following two panels show two independent sequences from single cell clone number 1-12 (deletion noted and vertical box indicates nucleotide insertion); the next two panels show two independent sequences from clone number 4-2 which has the same mutations described in clone 1-12; the final two panels show two independent sequences from clone number 4-5 which has a single nucleotide insertion (vertical box) and 7 nucleotide deletion. **B.** RT-PCR and BioAnalyzer gel-like image of RNA extracted from UD *NKX2.5*→EGFP hESCs described (see Figs 3A and 3B) above to measure alternative exon inclusion for *PICALM* (result shown is representative of 3 biological replicates with mean percent included +/- standard deviation shown below; each KO value is ****P < 0.0001 by Student's t-test). **C.** RT-PCR and agarose gel analysis of RNA extracted from UD *NKX2.5*→EGFP hESCs as described above to measure exon inclusion for *DNMT3B* (result shown is representative of 3 biological replicates). **D.** Western blot of 3 independent biological replicates of protein extracted from UD H9 hESCs, transduced with shNT, shQKI1, and shQKI2 probed with antibodies for panQKI (green) and GAPDH (magenta), and showing panQKI protein abundance relative to GAPDH in bar graph below, normalized to shNT (**$P \leq 0.01$ and ***$P \leq 0.001$ by Student's t-test). **E.** RT-PCR and BioAnalyzer gel-like image of RNA extracted from UD H9 hESCs transduced with shRNAs described above (in biological triplicate) to measure alternative exon inclusion of *RAI14*, *BIN1*, *CLSTN1*, and *NF1* (the mean values +/- standard deviation are reported below; *P < 0.05, **P < 0.01, ***P < 0.001, ****P < 0.0001 by Student's t-test). **F.** RT-PCR and BioAnalyzer gel-like image of RNA extracted from UD H9 hESCs transduced in triplicate with shRNAs described above (see Fig 3E) to measure alternative exon inclusion of *PICALM* (the mean values +/- standard deviation are reported below; ns denotes not significant, *P < 0.05). **G.** RT-PCR and agarose gel analysis of RNA extracted from UD H9 hESCs transduced in triplicate with shRNAs described above to measure exon inclusion of *DNMT3B*. H. RT-PCR and agarose gel of RNA extracted from UD *NKX2.5*→EGFP dCas9:VPR hESCs (described in Figs 3C and 3D) to measure exon inclusion for *DNMT3B*.

SUPPLEMENTAL FIGURE 4: A. RT-qPCR measuring abundance of RNA extracted from *NKX2.5*→EGFP UD hESCs, d4 CM cells, and d8 c.myo cells for *ISL1*, *GATA4*, *HAND1*, and *MEF2C* mRNAs relative to *HMBS* mRNA in WT, polyKO, or 4-2KO cells (biological replicate n = 2 or 3 each; ns = not significant, **P* ≤ 0.05, ***P* ≤ 0.01, ****P* ≤ 0.001 measured by Student's t-test). **B.** Indirect immunofluorescence of UD *NKX2.5*→GFP WT (left) or polyclonal *QKI*-KO (right) hESCs analyzed by high content imaging, showing QKI5 (magenta), OCT4 (yellow), EGFP (green), brightfield (gray), and DAPI (blue) with each fluorescent channel overlaid. On the right, the fluorescence intensity values per cell (relative to DAPI) are shown for QKI5 and OCT4 with *P* values calculated by Mann-Whitney U. **C.** Indirect immunofluorescence of *NKX2.5*→GFP WT (left) or polyclonal *QKI*-KO (right) d4 CM cells analyzed by high content imaging, showing panQKI (magenta), endogenous NKX2.5 (yellow), EGFP (green), brightfield (gray), and DAPI (blue) with each fluorescent channel overlaid. On the right, the fluorescent intensity values per cell (relative to DAPI) are shown for panQKI and NKX2.5 with *P* values calculated by Mann-Whitney U. **D.** Fluorescent intensity values per well measured by indirect immunofluorescence as described in Fig 4F for QKI5 and cTNT (TNNT2), with each normalized to DAPI, in WT and polyclonal QKI-KO day 8 c.myo *NKX2.5*→GFP cells (n = 3 independent biological replicates; ****P* ≤ 0.001 by Student's t-test). **E.** Low magnification epifluorescence imaging with FITC filter of live d8 c.myo *NKX2.5*→EGFP cells WT, polyKO, and 4-2KO (scale bar denotes 200 µm). **F.** Mean percent included values for alternatively spliced exons in *RAI14*, *NF1*, and *BIN1* calculated from three independent biological replicates, +/- standard deviation (see Fig 4H).

SUPPLEMENTAL FIGURE 5: A. UCSC Genome Browser screen shot showing QKI eCLIP peaks from K562 cells, Qk iCLIP peaks from C2C12 myoblasts, Gencode Genes with annotated *BIN1* transcripts, unique full-length *BIN1* cDNA clones sequenced (Sanger sequencing) from UD and d8 c.myo WT and *QKI*-KO *NKX2.5*→EGFP cells (KO11 represents one full-length clone from two different forward and reverse sequencing reactions), coverage tracks from UD hESCs, DE cells, CM cells, and ECT cells RNA-seq datasets, and conservation tracks. **B.** RT-qPCR of RNA extracted from *NKX2.5*→EGFP WT, polyKO, or 4-2KO UD hESCs, d4CM cells, or d8 c.myo cells, and measuring either the 5' end of *BIN1* (primer pair spanning exons 4 and 5) or the 3' end of *BIN1* (primer pair spanning exons 19 and 20); value shown is ddCt relative to *HMBS*; n = 2 or 3 independent replicates, ns = not significant, *$P < 0.05$, **$P < 0.01$, ***$P < 0.001$ by Student's t-test compared to WT. **C.** BIN1 infrared protein signal relative to GAPDH infrared protein signal from western blots shown in Figs 4C, 4G, and 4K (n = 3 independent replicates, ns = not significant, *$P < 0.05$, ***$P < 0.001$ by Student's t-test compared to WT). **D.** BIN1 protein abundance (as normalized exclusive intensity on y-axis) from WT UD hESC, WT d4 CM, and WT d8 c.Myo cell extracts measured by data-independent acquisition LC-MS/MS. **E.** BIN1 fluorescence intensity relative to GAPDH per well (n = 2), measured by high content imaging in UD, d4 CM, and d8 c.myo *NKX2.5*→EGFP WT, polyKO, or 4-2KO cells (ns = not significant, *$P < 0.05$, **$P < 0.01$ by Student's t-test compared to WT). **F.** BIN1 fluorescence intensity relative to GAPDH per cell (n = ~50,000 cells, per cell type) in d8 c.myo *NKX2.5*→EGFP WT, polyKO, or 4-2KO cells (****$P < 0.0001$ by Mann Whitney U). **G.** Mean percent inclusion (+/- standard deviation) values observed for RT-PCR and BioAnalyzer analysis shown in Figure 5 panels E, I, and M.

SUPPLEMENTAL FIGURE 6: A. UCSC Genome Browser screen shot spanning BIN1 exons 6 through 8 (top) or zoomed-in (bottom) at the 3'end of BIN1 alternatively spliced exon 7, showing panQk iCLIP reads from C2C12 myoblasts (top), Gencode gene annotation (middle), and PhyloP conservation tracks (bottom). The lower inset shows genomic DNA sequences with boxes around the exonic ACTAAC and intronic CTAAC sequences of interest. **B.** Agarose gel showing PCR amplification of the samples used in Fig 6C either with (+) or without (-) reverse transcription (top), and mean values (+/- standard deviation) of PCR products shown in Fig 6C obtained by RT-PCR and BioAnalyzer quantification (bottom).
