# Supplementary material for: Definition of germ layer cell lineage alternative splicing programs reveals a critical role for Quaking in specifying cardiac cell fate
Source: Nucleic Acids Res. 2022 May 11;50(9):5313–34. doi: 10.1093/nar/gkac327 (PMC9122611; doi:10.1093/nar/gkac327)
Supplement: gkac327_Supplemental_Files [file gkac327_supplemental_files.zip › SupplementalTable-Captions_NAR.docx]

**Supplemental Table descriptions**

**Supplemental Table S1:** DESEQ2 output including columns for gene, description, uniport accession, RBP/NA? (if annotated RBP, then value of “RBP” is listed, if it is not, “na” is listed), Ensembl gene ID, gene name, baseMean value, then log2 fold change and adjusted P-value for CM relative to UD, DE relative to UD, ECT relative to UD, and CM relative to DE, respectively.

**Supplemental Table S2:** VAST-tools output including columns for gene, event, then change in inclusion ratio (E[dPsi]) and the minimum predicted value of the change in inclusion ratio at 95% confidence interval (MV[dPsi]_at_0.95) for ECT relative to UD, CM relative to DE, CM relative to UD, or DE relative to UD.

**Supplemental Table S3:** rMATS output for cassette exons consisting of default column headers plus change in percent included calculated by VAST-tools (vastTools.dPSI) and whether a given exon was called as significantly differentially skipped or included (vastTools.signif), for ECT relative to UD, DE relative to UD, CM relative to UD, or DE relative to CM.

**Supplemental Table S4:** RBP-RELI output from analysis of exons alternatively spliced (called significant in both rMATS and VAST-tools) in CM compared to DE. Each of the first six tabs show the output from each region (AltEX is within the exon, DNintr is downstream intron, and UPintr is upstream intron) analyzed and whether more inclusion (INCL) or skipping (SKIP) were observed; the rows highlighted in blue indicate datasets that passed statistical significance cutoff for more skipping, and the rows highlighted in yellow indicate RBP datasets that passed statistical significance cutoff for more inclusion. The next two tabs (“merged”) show data for RBPs independent of region (UPintr, AltEX, DNintr) and was performed to calculate the total “ratio” score for RBP binding associated with either skipping or inclusion, which was used to determine the percentage of alternatively spliced events that showed evidence of binding by each RBP. The final tab shows the total sum of significantly observed QKI datasets’ binding to alternatively spliced exons in CM relative to DE. See also Methods for more information.

**Supplemental Table S5:** rMAPS2 output from rMATS “JCEC” cassette exon analysis of CM relative to DE. The data set on the left is for exons with significantly more skipping compared to background, and the data set on the right is for exons with significantly more inclusion compared to background.

**Supplemental Table S6:** LC-MS/MS data from UD, DE, and CM cells. RBP column is to denote if a protein is annotated as an RBP (+) or not (-).

**Supplemental Table S7:** LC-MS/MS DIA data from UD, CM, and d8 cardiomyocytes.

**Supplemental Table S8:** Oligonucleotide sequences used in the study, and their purposes.
